# Multiple Ribosomal RNA Operons in Bacteria; Their Concerted Evolution and Potential Consequences on the Rate of Evolution of Their 16S rRNA

**DOI:** 10.3389/fmicb.2018.01232

**Published:** 2018-06-08

**Authors:** Romilio T. Espejo, Nicolás Plaza

**Affiliations:** ^1^Institute of Nutrition and Food Technology, Universidad de Chile, Santiago, Chile; ^2^Centro de Investigación Biomédica, Facultad de Ciencias de la Salud, Instituto de Ciencias Biomédicas, Universidad Autónoma de Chile, Santiago, Chile

**Keywords:** 16S rRNA, *rrs*, multiple-copies, polymorphism, evolution, concerted

## Abstract

Bacterial species differ greatly in the number and location of the rRNA operons which may be present in the bacterial chromosomes and plasmids. Most bacterial species contain more than one ribosomal RNA operon copy in their genomes, with some species containing up to 15 such copies. We review the number and location of the rRNA operons and discuss evolution of 16S rRNA (*rrs*) genes -which are considered as ultimate chronometers for phylogenetic classification- in bacteria with multiple copies of these genes. In these bacterial species, the *rrs* genes must evolve in concert and sequence changes generated by mutation or horizontal gene transfer must be either erased or spread to every gene copy to avoid divergence, as it occurs when they are present in different species. Analysis of polymorphic sites in intra-genomic *rrs* copies identifies putative conversion events and demonstrates that sequence conversion is patchy and occurs in small conversion tracts. Sequence conversion probably arises by a non-reciprocal transfer between two or more copies where one copy contributes only a small contiguous segment of DNA, whereas the other copy contributes the rest of the genome in a fairly well understood molecular process. Because concerted evolution implies that a mutation in any of the *rrs* copies is either eliminated or transferred to every *rrs* gene in the genome, this process should slow their evolution rate relative to that of single copy genes. However, available data on the *rrs* genes in bacterial genomes do not show a clear relationship between their evolution rates and the number of their copies in the genome.

## Ribosomal Rna Operons in Bacteria

**FIGURE 1:**
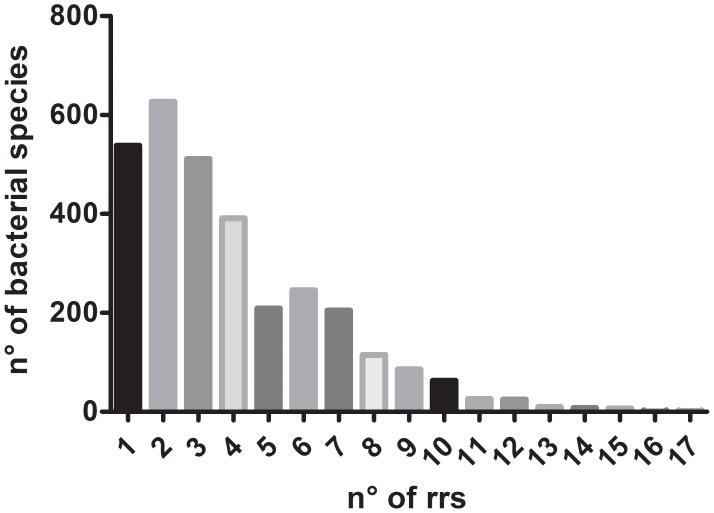
Median of the number of *rrs* copies in 3,070 bacterial species according to data reported in *rrn*DB database (Stoddard et al., 2015).

In bacteria, the 5S, 16S, and 23S rRNA genes are organized into a gene cluster linked together by internal transcribed spacer (ITS) regions containing tRNAs and conserved adjacent regions. The cluster is expressed as a single operon, and the individual RNA molecules transcribed are processed by at least three different RNAases into rRNAs and tRNAs (Apirion and Miczak, 1993). The number and location of rRNA operons (*rrn*) is very diverse: they may be present in 1–15 copies in prokaryotic genomes (Schmidt, 1997; Klappenbach et al., 2001; Pei et al., 2010) and over 80% bacterial genomes sequenced have more than one operon. Recently, the presence of 17 *rrs* was reported in *Paeniclostridium sordellii* CBA7122, a species with genomes containing 1 to 8 rRNA operons (Kim et al., 2017). **Figure 1** shows the median of the number of *rrs* copies in 3,070 bacterial species according to data reported in *rrn*DB database (Stoddard et al., 2015; **Supplementary Table S1** show the data in detail). Multiple *rrn* copies would result in a selective pressure to maintain and rapidly increase high ribosome content that would enable rapid adaptation to nutritional upshift or favorable temperature change (Klappenbach et al., 2000; Roller et al., 2016). Multiple rRNA operons are not essential: *E. coli* remains viable with just one out of its seven operons (Asai et al., 1999b). Moreover, its function can be replaced by a plasmid-encoded rRNA operon, which in *E. coli* can be exchanged for operons from *Salmonella typhimurium* and *Proteus vulgaris* (Asai et al., 1999a). However, bacterial species with multiple rRNA operons that survive with a single operon show a decreased fitness (Stoddard et al., 2015). The number of ribosomal operons allows predicting growth rate and growth efficiency –defined as Carbon use efficiency (biomass C/utilized C)- (Roller et al., 2016). It seems that the *rrn* number is the result of the physical and biological environment where the species –or the strain- thrive (Stevenson and Schmidt, 2004).

The number of *rrn* is independent of bacterial genome size: for example, the *Bradyrhizobium diazoefficiens* genome of 9.1 Mb contains only one *rrn* copy (Kaneko et al., 2002), whereas the genomes of *E. coli* (4.6 Mb) and *Bacillus subtilis* (4.2 Mb) contain seven and ten copies, respectively (Kiss et al., 1977; LaFauci et al., 1986). Furthermore, *rrn* copies may be present in more than one chromosome in bacteria that possess two chromosomes, e.g., in *Brucella* (Michaux et al., 1993) and *Vibrio* (Yamaichi et al., 1999). In addition, *rrn* copies are also found in both chromosomes and plasmids as in *B. megaterium* (Kunnimalaiyaan et al., 2001) or *Paracoccus* species (Battermann et al., 2003). A special case is *Aureimonas* sp. AU20 in which the only rRNA operon is in the plasmid (Anda et al., 2015).

## Concerted Evolution of rRna Genes

Because of its essential function, ubiquity, and evolutionary properties, the 16S rRNA gene (*rrs*) has become the most commonly used molecular marker in microbial ecology. It has long been utilized as an “ultimate chronometer” for phylogenetic classification of bacterial species (Woese, 1987). Evolution of *rrs* was originally thought to occur almost exclusively by vertical transmission of mutations because it was assumed that recombination with horizontally transferred DNA was precluded by the co-evolution of rRNA with many other components in the translational machinery (Jain et al., 1999), but see also work of Lawrence and other publications (Asai et al., 1999b; Lawrence, 1999; Acinas et al., 2004). However, *E. coli rrs* can be effectively replaced by foreign rRNA operons derived, for example, from *S. typhimurium* or *P. vulgaris* (Asai et al., 1999b). Furthermore, a comparison of *rrs* sequences between closely related species and between *rrs* copies in the same genome indicated that as with most other genes, recombination with horizontally transferred *rrs* is relatively frequent (reviewed by Kitahara and Miyazaki, 2013). Comparison of the sequences among *rrs* copies in the same genome has shown a frequent occurrence of short segments containing an abnormally high number of non-random base variations, which are thought to originate from the recombination of short segments of *rrs* with horizontally transferred DNA that contains segments of 16S rRNA gene (Cilia et al., 1996; Wang and Zhang, 2000). This finding is further supported by the observation that the difference between these polymorphic segments, besides the abnormal accumulation of substitutions, includes compensatory changes that maintain 16S rRNA secondary structure, implying that the divergence is relatively ancient and that each version evolved in different bacteria. This type of divergence has been reported in many unrelated species (Sneath, 1993; Yap et al., 1999; Moreno et al., 2002; González-Escalona et al., 2005; Morandi et al., 2005; Michon et al., 2010; Bodilis et al., 2012). Recently, analysis of 2,143 genomes distinguished 28 genomes with high intragenomic heterogeneity revealing horizontal gene transfer (HGT) events and their potential donors (Tian et al., 2015). In all these 28 cases HGT of the 16S rRNA gene only occurred at intrageneous or intraspecies levels. More recently, a conserved, highly divergent *rrs* (7.3–9.0%) was found in one of the five ribosomal operons in the species complex *Scytonema hyalinum* (Johansen et al., 2017).

In bacteria containing more than one *rrn*, polymorphisms between intragenomic *rrs* are frequently found (Cilia et al., 1996; Acinas et al., 2004). These polymorphisms can lead to the overestimation of bacterial diversity when it is assessed from the number and class of different 16S rRNAs in total DNA or RNA extracts (Pei et al., 2010). Detection of these polymorphic sites requires, however, a particular care: direct sequencing of PCR product of whole DNA produces a mean sequence where the polymorphism may remain hidden: high throughput sequencing renders short reads which cannot be unequivocally assigned to a particular *rrs* in the genome. To detect the polymorphic sites more reliably, intragenomic *rrs* should be independently sequenced or properly assembled from long reads obtained by high throughput sequencing that would allow specific assignment of the read to each particular *rrs*. Polymorphisms within the *rrs* can be directly uncovered by a simple procedure consisting of PCR amplification and observation of the formation of *rrs* heteroduplexes that migrate slower than the homoduplex during electrophoresis in polyacrylamide gel (Moreno et al., 2002). Polymorphic sites in intragenomic *rrs* are, however, scarce and occur at a much lower frequency than between *rrs* of different species. For this reason, it is thought that multiple *rrs* copies in the same genome evolve in concert. Concerted evolution converts multiple copies of a gene in a multigene family into copies with identical or similar sequences. It was first proposed in *E. coli* and *H. influenzae*, where average divergence is 0.0055 and 0 per site among the seven *rrs* genes in *E. coli* and six *rrs* genes in *H. influenzae*, respectively. At the same time, the average interspecies divergence between the *rrs* genes of *E. coli* and *H. influenzae* is 0.1325 (Liao, 2000). Detailed observation of the distribution of the polymorphic sites among the *rrs* genes of the *E. coli* strain used in that study indicated that several sequence conversion events had taken place and that the conversion tracts were smaller than 500 bp. Liao concluded “that sequence conversion between paralogous rRNA genes is patchy and occurs in discontinuous tracts throughout the genic regions.” We will avoid using the term “paralogous” for *rrs* genes in the same genome because they do not strictly correspond to the established definition of paralogous genes: “Related genes that have resulted from a gene duplication event within a single genome and are likely to have diverged in their function are said to be paralogs” (Alberts et al., 2002). Cilia et al. (1996) arrived at a similar conclusion, having compared the divergence of each operon in *E. coli* and *S. typhimurium*: “most point mutations that occurred within each gene have been propagated among the gene family by conversions involving short domains, and … homogenization by conversions may not have affected the entire sequence of each gene.” In contrast, a study of gene conversion between the seven rRNA operons in an *E. coli* mutant led to different conclusions, which was probably due to the particular experimental system employed (Hashimoto et al., 2002). Hashimoto et al. built a mutant with one *rrs*, which contained a cassette harboring genes responsible for sucrose sensitivity and neomycin resistance, and measured the replacement of this modified *rrs* by a non-modified *rrs*. They observed that the complete *rrs* gene was restored at a rate of 5 × 10^-9^ (per cell division), i.e., a rate too low to erase mutations because during concerted evolution, the conversion rate has to be higher than the mutation rate. Hashimoto et al. (2002) also observed in some occasions the restoration of the whole operon, including replacement of the ITS region between the *rrs* and 23S rRNA (*rrl*), implying that recombination involved a large piece of the *rrn* operon. However, both this last observation and the assessed rate of conversion may be a consequence of the particular experimental system created to study conversion, namely the *rrs* gene with a large insertion. It seems more likely that conversion occurs at a higher rate than that observed in this particular mutant and in short pieces. Homogenization of multiple *rrs* sequences is, however, not absolutely effective, and the number of polymorphic sites in the *rrs* genes results from the equilibrium between the rate of homogenization by concerted evolution and the rate of differentiation by mutation and recombination with exogenous *rrs* genes.

Polymorphic sites could have a functional role, for example a mutation in 16S rRNA of *Enterococcus faecium* decreases binding of aminoglycosides to the 30S small ribosomal subunit changing its intrinsic resistance to this antibiotic (Galimand et al., 2011). A similar observation was made for the 23S rRNA by Werner et al. (2007) and they further showed that the presence of a single mutated 23S rRNA gene allele among the four *rrs* copies in this species (Stoddard et al., 2015) leads to phenotypically detectable resistance in some isolates. They speculated that 23S rRNA gene alleles could be expressed heterogeneously, which might influence the amount of replicated mutated rRNA available for formation of resistant ribosomes.

## Molecular Mechanism of Concerted Evolution

**FIGURE 2:**
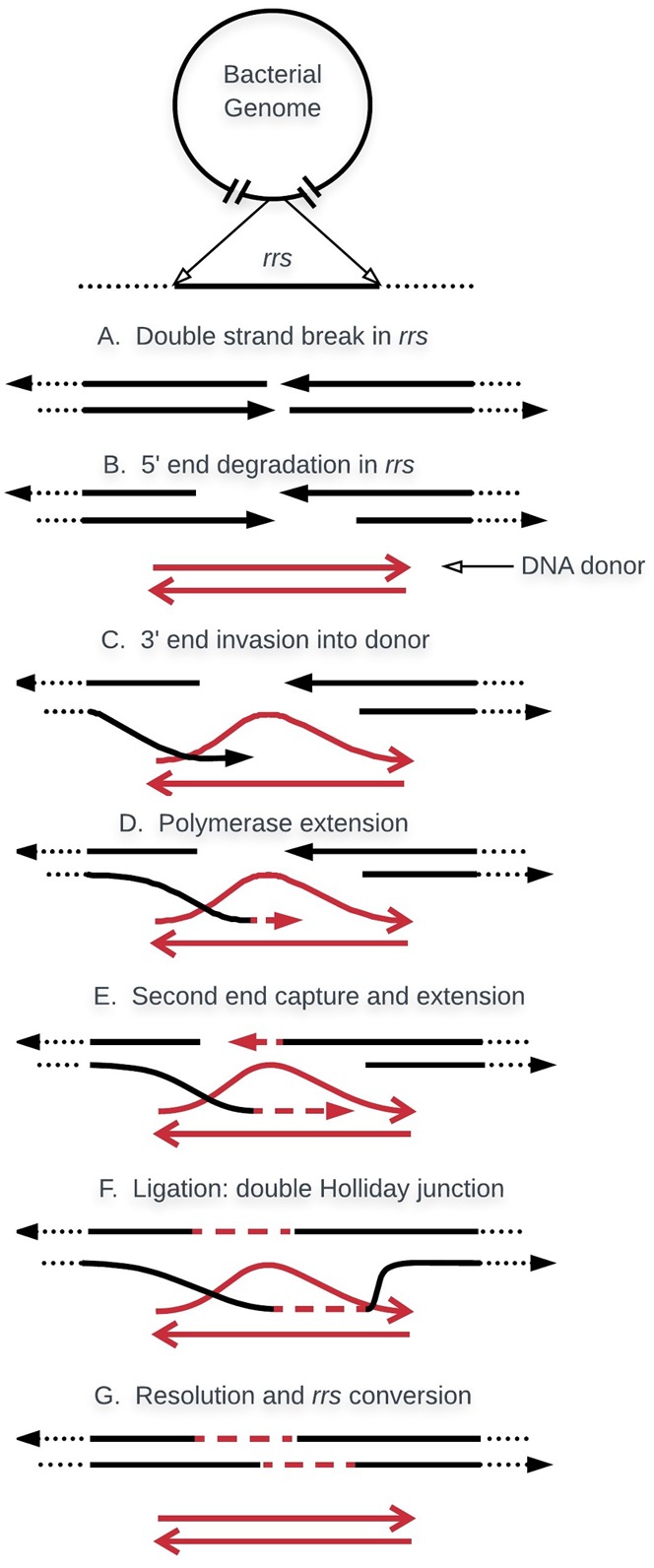
Basic model for gene conversion. In bacteria with multiple copies, *rrs* evolve in concert by non-reciprocal recombination, where one copy contributes the genome while the other copy provides only a segment of the *rrs*, or the whole gene. The process is initiated by a DSB in the *rrs* of the genome (red). This is processed by RecBCD exonuclease/helicase to create a gap and an ssDNA (arrow heads are 3^′^-ends). RecA promotes strand invasion of the *rrs* segment. The invading tail then initiates new DNA synthesis which extends until it overlaps and anneals with the other end of the DSB. Through a similar process, the displaced DNA strand forms hDNA with the second 3^′^-end ssDNA. Two Holliday Junctions are formed which are able to migrate and extend hDNA. Segments of gene conversion can be generated in two different ways. One alternative is through repair, by DNA synthesis, of the gap produced during initiation. The other is through the methyl- directed mismatch repair system which may “repair” hDNA by destroying one strand of DNA in the heteroduplex, followed by new DNA synthesis using the remaining strand as template.

The molecular mechanism of gene conversion in bacteria likely occurs by a non-reciprocal transfer between two or more copies where one copy contributes only a small contiguous segment of DNA, whereas the other contributes the rest of the genome (Santoyo and Romero, 2005). The most widely accepted models that explain this type of recombination focus on double-strand break repair (Orr-Weaver et al., 1988; Hastings, 2010) and synthesis-dependent strand annealing (Ira et al., 2003). **Figure 2** illustrates *rrs* gene conversion according to the two models cited above. A similar process seems to take place during gene conversion of the duplicated *tuf* genes in *Salmonella* (Paulsson et al., 2017). Most of the proteins that play essential roles in this non-reciprocal recombination, such as those participating in annealing and strand exchange (*recA*), migration, and resolution of the Holliday junction (*ruv*A, *ruv*B, *ruv*C, and *recG*), have been described in *E. coli* (Kowalczykowski et al., 1994).

Recombination can effectively act in concerted evolution if it is non-reciprocal and occurs without interrupting or disorganizing the genome as shown in **Figure 2**. These features can be accomplished if recombination occurs between the genome and DNA segments. These DNA segments could be either generated internally or by horizontal transfer. A high rate of intergenomic *rrs* recombination was shown in *Vibrio parahaemolyticus* and the authors postulated that this could be an effective mechanism to maintain intragenomic *rrs* cohesion (Harth et al., 2007): if the incoming DNA contains the prime sequence among the *rrs* genes, it will reconvert any mutant *rrs* to the main (most abundant) sequence. This may happen when clonal bacteria are in close contact, which occurs in colonies or biofilms. After studying the intragenomic heterogeneity of the *rrs* genes in *Vibrio* strains, authors concluded, “This high *rrs* homologous intergenomic recombination could be an effective mechanism to maintain intragenomic *rrs* cohesion, mediating the dispersal of the most abundant *rrs* version in the clone among the eleven intragenomic loci” (Harth et al., 2007). The *rrs* composition observed within a clonal population will be maintained as long as intergenomic recombination occurs among the members of the same clone at a rate higher than mutation rate. However, there is no way to detect and measure recombination between identical genes. Nonetheless, it is accepted that recombination increases with nucleotide similarity and is higher among identical genes (Majewski and Cohan, 1999). Furthermore, it has been shown that intergenomic recombination between highly homologous genes may be more frequent than mutations (Feil et al., 1999). However, little is known on the dynamics of recombination, for example whether every *rrs* recombines at the same rate, what are the lengths of the conversion tracts, what is the extent of recombination between clonal bacteria, and to what extent conversion is influenced by other components and events.

## Consequence of Concerted Evolution on the Rates of *rrs* Evolution

Based on the assumption that substitution rates are fairly constant through time and across taxa, differences in the sequences of core genes have been employed as molecular clocks to estimate divergence times in bacteria (Battistuzzi et al., 2004; Kuo and Ochman, 2009). In particular, 16S rRNA sequence has long been used as an “ultimate chronometer” for phylogenetic classification of bacterial species (Woese, 1987). The absolute rate of 16S rRNA divergence in eubacteria, calculated by linking the appearance of several bacterial lineages to events that occurred at known times in the geologic past, was estimated to be 0.02% per million years (Ochman and Wilson, 1987). However, the calibration of these molecular clocks showed variable results: the rate of 16S rRNA sequence divergence in obligate endosymbionts calibrated by the fossil records of their host insects varied from 0.025 to 0.091% per million years, i.e., approximately four-fold, across the six lineages included in a recent study (Kuo and Ochman, 2009). However, all these divergence rate estimates assume either the same generation time for all bacteria included in the study or figures that are difficult to verify. In a complementary approach, when divergence estimates based on a set of universally conserved protein-coding genes were compared with those based on 16S rRNA genes, a low correlation was observed (Kuo and Ochman, 2009): substitutions in 16S rRNA (*K*_16S_) exhibited low correspondence with the median non-synonymous (*K*_a_) or synonymous substitutions (*K*_s_) in the set of protein-coding genes across bacterial taxa. However, a low correlation of the substitution rates for the different protein-coding genes taken individually was noted: *K*_s_ values ranged from 0.11 to 1.29 (average = 0.6), and *K*_a_ values ranged from 0.006 to 0.058 (average = 0.03). Concerted evolution implies that mutations in one *rrs* gene are either erased or transferred to every *rrs* gene in the genome leading to the fixation of the mutation that occurred in one of the *rrs* genes. In *V. parahaemolyticus,* for example, fixation would require a transfer of the mutation in one *rrs* gene by non-reciprocal recombination to ten other *rrs* genes. The removal of mutations or their transfer to the rest of the *rrs* genes should slow the evolution rate of *rrs* in bacterial genomes possessing multiple rRNA operons. Notably, the rates of evolution of 16S rDNA sequences have been found to be significantly higher in endosymbionts (*Buchnera*) than in related bacteria *E. coli*/*S. enterica* (Moran and Clegg, 1996). The authors of the study attributed this difference to the endosymbiotic nature of *Buchnera* but they did not consider that *Buchnera* species contain a single *rrs*, whereas *E. coli*/*S. enterica* contain seven *rrs* genes. Substitutions in 16S rRNA (*K*_16S_) have been found to exhibit low correspondence with either *K*_a_ and *K*_s_ substitutions in a set of protein-coding single genes between pairs of bacterial strains across taxa (Kuo and Ochman, 2009). Pearson coefficient of the correlation *K*_a_ vs. *K*_16s_ values was 0.55. We explored whether the value of this Pearson correlation coefficient would increase when *K*_16S_ was multiplied by the number of the *rrs* genes in the respective bacterial pair, assuming that *K*_16S_ value would decrease with the number of *rrs*. However, our calculations yielded an even lower Pearson correlation coefficient of 0.29 for *K*_a_ vs. *K*_16S_ × No. of *rrs* genes. It is possible, however, that because the values of *K*_a_ and *K*_16S_ include nucleotide differences caused by spontaneous mutations and recombination, the correlation could be potentially higher if only spontaneous mutations were to be considered.

## Conclusion

Advances in this field are scarce since the last review of Santoyo and Romero (2005); with this review we expect to call attention to the need to advance on the concerted evolution of the 16S rRNA genes and its consequence on their evolution rate. 16S rRNA genes are present in different number and location in bacterial species and their number seems the result of the physical and biological environment where the species thrive. In bacteria with multiple copies, *rrs* evolve in concert by non-reciprocal recombination, where one copy contributes the genome while the other copy provides only a segment of the *rrs*, or the whole gene.

The copy contributing part of the genome may be acquired by horizontal transfer. 16S rRNA sequences have long been used as an “ultimate chronometer” for phylogenetic classification of bacterial species but the effect of multiple *rrs* in bacterial genomes and their location in chromosomes or plasmids has not been considered. Further understanding of concerted evolution of *rrs* when in multiple copies in the genome such as the exact mechanism of recombination, the rate of erasing or spreading of the original base change occurring in one *rrs*, and the possible difference on the rate of non-reciprocal recombination of the different *rrs* in the genome will help to detail its effect on the evolution of the 16S rRNA.

## Author Contributions

RE reviewed the literature and wrote the manuscript. NP extended revision of literature, revised data and drew figures, and collaborated in the writing of the manuscript.

## Conflict of Interest Statement

The authors declare that the research was conducted in the absence of any commercial or financial relationships that could be construed as a potential conflict of interest.
